# Cs_2_Bi(PO_4_)(WO_4_)

**DOI:** 10.1107/S160053680903147X

**Published:** 2009-08-15

**Authors:** Kateryna V. Terebilenko, Igor V. Zatovsky, Vyacheslav N. Baumer, Nikolay S. Slobodyanik

**Affiliations:** a Department of Inorganic Chemistry, Taras Shevchenko National University, 64 Volodymyrska Street, 01601 Kyiv, Ukraine; b STC ‘Institute for Single Crystals’, NAS of Ukraine, 60 Lenin ave., 61001 Kharkiv, Ukraine

## Abstract

Dicaesium bis­muth(III) phosphate(V) tungstate(VI), Cs_2_Bi(PO_4_)(WO_4_), has been synthesized during complex investigation in a molten pseudo-quaternary Cs_2_O–Bi_2_O_3_–P_2_O_5_–WO_3_ system. It is isotypic with K_2_Bi(PO_4_)(WO_4_). The three-dimensional framework is built up from [Bi(PO_4_)(WO_4_)] nets, which are organized by adhesion of [BiPO_4_] layers and [WO_4_] tetra­hedra above and below of those layers. The inter­stitial space is occupied by Cs atoms. Bi, W and P atoms lie on crystallographic twofold axes.

## Related literature

For the isotypic potassium analogue, see: Zatovsky *et al.* (2006 ▶). For a related structure, see: Terebilenko *et al.* (2008 ▶). For caesium coordination, see Borel *et al.* (2000 ▶); Yakubovich *et al.* (2006 ▶)

## Experimental

### 

#### Crystal data

Cs_2_Bi(PO_4_)(WO_4_)
*M* *_r_* = 817.61
Orthorhombic, 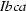

*a* = 21.3144 (10) Å
*b* = 12.6352 (5) Å
*c* = 7.1412 (3) Å
*V* = 1923.21 (14) Å^3^
*Z* = 8
Mo *K*α radiation
μ = 37.87 mm^−1^
*T* = 293 K
0.08 × 0.07 × 0.05 mm

#### Data collection

Oxford Diffraction XCalibur-3 diffractometer
Absorption correction: multi-scan (Blessing, 1995 ▶) *T*_min_ = 0.061, *T*_max_ = 0.174 (expected range = 0.053–0.151)
10697 measured reflections
1396 independent reflections
1227 reflections with *I* > 2σ(*I*)
*R*_int_ = 0.156

#### Refinement

*R*[*F* ^2^ > 2σ(*F* ^2^)] = 0.052
*wR*(*F* ^2^) = 0.115
*S* = 1.21
1396 reflections
61 parameters
Δρ_max_ = 2.17 e Å^−3^
Δρ_min_ = −2.63 e Å^−3^

### 

Data collection: *CrysAlis CCD* (Oxford Diffraction, 2006 ▶); cell refinement: *CrysAlis CCD*; data reduction: *CrysAlis RED* (Oxford Diffraction, 2006 ▶); program(s) used to solve structure: *SHELXS97* (Sheldrick, 2008 ▶); program(s) used to refine structure: *SHELXL97* (Sheldrick, 2008 ▶); molecular graphics: *DIAMOND* (Brandenburg, 1999 ▶); software used to prepare material for publication: *WinGX* (Farrugia, 1999 ▶) and *enCIFer* (Allen *et al.*, 2004 ▶).

## Supplementary Material

Crystal structure: contains datablocks global, I. DOI: 10.1107/S160053680903147X/br2113sup1.cif

Structure factors: contains datablocks I. DOI: 10.1107/S160053680903147X/br2113Isup2.hkl

Additional supplementary materials:  crystallographic information; 3D view; checkCIF report

## Figures and Tables

**Table 1:** Selected bond lengths (Å)

Bi1—O2	2.388 (8)
Bi1—O1^i^	2.389 (8)
Bi1—O3^ii^	2.463 (8)
Bi1—O1^iii^	2.669 (8)
Cs1—O2^ii^	2.990 (8)
Cs1—O4^iv^	3.031 (9)
Cs1—O2^i^	3.046 (8)
Cs1—O3^v^	3.088 (9)
Cs1—O4^vi^	3.111 (10)
Cs1—O4^vii^	3.140 (9)
Cs1—O1	3.338 (9)
Cs1—O3^vii^	3.339 (9)
W1—O4	1.774 (9)
W1—O3^vi^	1.792 (9)
P1—O1	1.539 (8)
P1—O1^ii^	1.539 (8)
P1—O2^ii^	1.549 (8)

Symmetry codes: (i) 
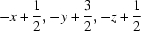
; (ii) 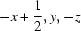; (iii) 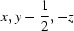; (iv) 
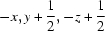
; (v) 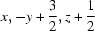; (vi) 
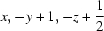
; (vii) 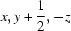.

**Fig. 1.**
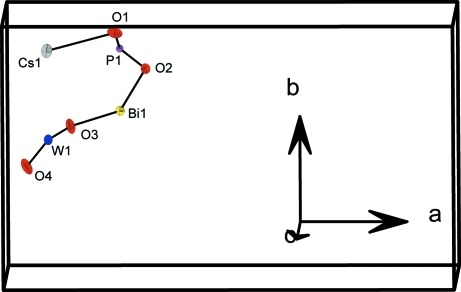
View of the title compound with displacement ellipsoids at the 50% probability level.

**Fig. 2.**
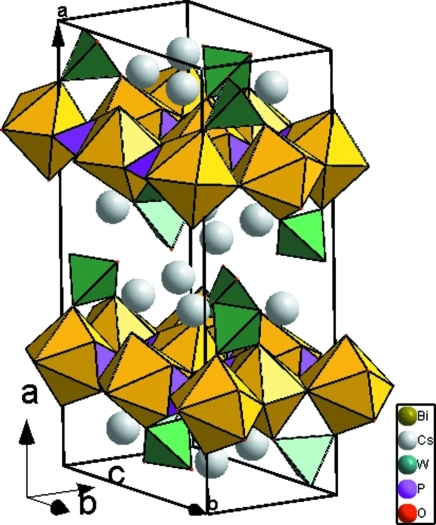
View of Cs2Bi(PO4)(WO4).

## supplementary crystallographic information

### Comment

Chemistry of caesium phosphates shows a great diversity due to its structural flexibility in adopting different coordination environment. In metal phosphates caesium resides generally in complex polyhedron with up to fourteen vertices providing formation of two- and three-dimensional frameworks. Depending on crystal structure caesium is believed to occupy big cavities and tunnels adapting their geometry. For instance, the structure of Cs_3_Mo_8_O_11_(PO_4_)_8_ (Borel *et al.*, 2000) represents two types of irregular surrounding with nine and ten oxygen coordination, Cs_2_Ti(VO_2_)_3_(PO_4_)_3_ (Yakubovich *et al.*,2006) - twelve and fourteen. Herein, the structure of K_2_Bi(PO_4_)(WO_4_) (Zatovsky *et al.*, 2006) represents an interesting host for substitution of potassium atoms by caesium ones, that leads to formation of the first example of caesium-containing phosphate-tungstate Cs_2_Bi(PO_4_)(WO_4_) (Fig 1). Three-dimensional framework of the title compound is organized by linking together [Bi(PO_4_)(WO_4_)] nets which are formed by adhesion [BiPO_4_] layers and WO_4_ tetrahedra above and below of those layers (Fig. 2). Both phosphate and tungstate tetrahedra have almost regular geometry with typical bond lengths. Caesium atom resides in interlayer space having eightfold coordination duplicating potassium ones' environment in the structure of K_2_Bi(PO_4_)(WO_4_) (Zatovsky *et al.*, 2006). Due to bigger ionic radius of Cs, the distance between two successive nets (a half of a cell dimension a) is 10.657 Å, while for K-analogue is 9.862 Å.

### Experimental

Single crystals of the title compound were obtained during investigation in the pseudo-quaternary molten system Cs_2_O—Bi_2_O_3_—P_2_O_5_—WO_3_. A mixture of CsPO_3_ (1.060 g), Cs_2_W_2_O_7_ (3.725 g) and Bi_2_O_3_ (0.840 g) were mixed in an agate mortar, and heated in a platinum crucible up to 1223 K to obtain a homogeneous melt. It was held at this temperature for an hour and cooled down with a rate of 40 K h^-1^ to 833 K. Crystalline product was leached out from the solidified melt with hot water.

### Refinement

Convergence factors (R, wR) and R_int_ are high due to low intensity of the reflections which is connected with poor quality of crystals. Experiments were carried out for several crystals from different synthetic points, unfortunately, better results than is presented were not found. Taking into account the previous structures isotypic to titled compound there is no doubts in structure determination.

The highest peak and the deepest hole in the final difference map are located at 0.77Å from P1 (2.173 e/Å^3^) and 0.70Å from P2 (-2.633 e/Å^3^) respectively.

### Crystal data

**Table:**

Cs_2_Bi(PO_4_)(WO_4_)	*F*(000) = 2768
*M**_r_* = 817.61	*D*_x_ = 5.648 Mg m^−^^3^
Orthorhombic, *I**b**c**a*	Mo *K*α radiation, λ = 0.71073 Å
Hall symbol: -I 2b 2c	Cell parameters from 10697 reflections
*a* = 21.3144 (10) Å	θ = 3.2–30.0°
*b* = 12.6352 (5) Å	µ = 37.87 mm^−^^1^
*c* = 7.1412 (3) Å	*T* = 293 K
*V* = 1923.21 (14) Å^3^	Prism, colourless
*Z* = 8	0.08 × 0.07 × 0.05 mm

### Data collection

**Table:**

Oxford Diffraction XCalibur-3 diffractometer	1396 independent reflections
Radiation source: fine-focus sealed tube	1227 reflections with *I* > 2σ(*I*)
graphite	*R*_int_ = 0.156
φ and ω scans	θ_max_ = 30.0°, θ_min_ = 3.2°
Absorption correction: multi-scan (Blessing, 1995)	*h* = −27→29
*T*_min_ = 0.061, *T*_max_ = 0.174	*k* = −17→17
10697 measured reflections	*l* = −10→10

### Refinement

**Table:**

Refinement on *F*^2^	0 restraints
Least-squares matrix: full	Primary atom site location: structure-invariant direct methods
*R*[*F*^2^ > 2σ(*F*^2^)] = 0.052	Secondary atom site location: difference Fourier map
*wR*(*F*^2^) = 0.115	*w* = 1/[σ^2^(*F*_o_^2^) + (0.0396*P*)^2^ + 22.3992*P*] where *P* = (*F*_o_^2^ + 2*F*_c_^2^)/3
*S* = 1.21	(Δ/σ)_max_ < 0.001
1396 reflections	Δρ_max_ = 2.17 e Å^−^^3^
61 parameters	Δρ_min_ = −2.63 e Å^−^^3^

### Special details

**Table:**

Geometry. All e.s.d.'s (except the e.s.d. in the dihedral angle between two l.s. planes) are estimated using the full covariance matrix. The cell e.s.d.'s are taken into account individually in the estimation of e.s.d.'s in distances, angles and torsion angles; correlations between e.s.d.'s in cell parameters are only used when they are defined by crystal symmetry. An approximate (isotropic) treatment of cell e.s.d.'s is used for estimating e.s.d.'s involving l.s. planes.
Refinement. Refinement of *F*^2^ against ALL reflections. The weighted *R*-factor *wR* and goodness of fit *S* are based on *F*^2^, conventional *R*-factors *R* are based on *F*, with *F* set to zero for negative *F*^2^. The threshold expression of *F*^2^ > σ(*F*^2^) is used only for calculating *R*-factors(gt) *etc*. and is not relevant to the choice of reflections for refinement. *R*-factors based on *F*^2^ are statistically about twice as large as those based on *F*, and *R*- factors based on ALL data will be even larger.

### Fractional atomic coordinates and isotropic or equivalent isotropic displacement parameters (Å^2^)

**Table:**

	*x*	*y*	*z*	*U*_iso_*/*U*_eq_	
Bi1	0.25	0.58662 (4)	0	0.01381 (17)	
Cs1	0.09029 (3)	0.83471 (6)	0.21999 (11)	0.0233 (2)	
W1	0.09279 (3)	0.5	0.25	0.01566 (18)	
P1	0.25	0.8232 (3)	0	0.0081 (6)	
O1	0.2413 (4)	0.8984 (6)	0.1675 (11)	0.0204 (17)	
O2	0.3072 (4)	0.7487 (6)	0.0220 (11)	0.0173 (15)	
O3	0.1403 (4)	0.5328 (8)	0.0513 (13)	0.0258 (18)	
O4	0.0440 (4)	0.3925 (8)	0.1847 (13)	0.031 (2)	

### Atomic displacement parameters (Å^2^)

**Table:**

	*U*^11^	*U*^22^	*U*^33^	*U*^12^	*U*^13^	*U*^23^
Bi1	0.0116 (3)	0.0135 (3)	0.0162 (3)	0	−0.00033 (18)	0
Cs1	0.0166 (4)	0.0290 (4)	0.0243 (4)	0.0014 (3)	−0.0003 (2)	0.0005 (3)
W1	0.0107 (3)	0.0162 (3)	0.0201 (3)	0	0	0.0007 (2)
P1	0.0077 (15)	0.0086 (13)	0.0080 (15)	0	−0.0014 (10)	0
O1	0.034 (5)	0.015 (3)	0.012 (4)	−0.004 (3)	0.002 (3)	−0.003 (3)
O2	0.013 (4)	0.017 (3)	0.021 (4)	−0.001 (3)	0.000 (3)	0.003 (3)
O3	0.013 (4)	0.036 (5)	0.028 (4)	−0.007 (4)	−0.004 (3)	0.006 (4)
O4	0.020 (5)	0.032 (5)	0.040 (5)	−0.013 (4)	0.007 (4)	−0.013 (4)

### Geometric parameters (Å, °)

**Table:**

Bi1—O2	2.388 (8)	Cs1—O4^viii^	3.111 (10)
Bi1—O2^i^	2.388 (8)	Cs1—O4^ix^	3.140 (9)
Bi1—O1^ii^	2.389 (8)	Cs1—O1	3.338 (9)
Bi1—O1^iii^	2.389 (8)	Cs1—O3^ix^	3.339 (9)
Bi1—O3^i^	2.463 (8)	W1—O4	1.774 (9)
Bi1—O3	2.463 (8)	W1—O4^viii^	1.774 (9)
Bi1—O1^iv^	2.669 (8)	W1—O3^viii^	1.792 (9)
Bi1—O1^v^	2.669 (8)	W1—O3	1.792 (9)
Cs1—O2^i^	2.990 (8)	P1—O1	1.539 (8)
Cs1—O4^vi^	3.031 (9)	P1—O1^i^	1.539 (8)
Cs1—O2^ii^	3.046 (8)	P1—O2^i^	1.549 (8)
Cs1—O3^vii^	3.088 (9)	P1—O2	1.549 (8)
			
?—?—?	?		

Symmetry codes: (i) −*x*+1/2, *y*, −*z*; (ii) −*x*+1/2, −*y*+3/2, −*z*+1/2; (iii) *x*, −*y*+3/2, *z*−1/2; (iv) *x*, *y*−1/2, −*z*; (v) −*x*+1/2, *y*−1/2, *z*; (vi) −*x*, *y*+1/2, −*z*+1/2; (vii) *x*, −*y*+3/2, *z*+1/2; (viii) *x*, −*y*+1, −*z*+1/2; (ix) *x*, *y*+1/2, −*z*.
